# Childhood Asthma: Diagnosis and Treatment

**DOI:** 10.6064/2012/674204

**Published:** 2012-12-13

**Authors:** Wim M. van Aalderen

**Affiliations:** Department of Pediatric Respiratory Disease and Allergy, Emma Children's Hospital AMC, Meibergdreef 7, 1105 AZ Amsterdam, The Netherlands

## Abstract

Many children suffer from recurrent coughing, wheezing and chest tightness. In preschool children one third of all children have these symptoms before the age of six, but only 40% of these wheezing preschoolers will continue to have asthma. In older school-aged children the majority of the children have asthma. Quality of life is affected by asthma control. Sleep disruption and exercised induced airflow limitation have a negative impact on participation in sports and social activities, and may influence family life. The goal of asthma therapy is to achieve asthma control, but only a limited number of patients are able to reach total control. This may be due to an incorrect diagnosis, co-morbidities or poor inhalation technique, but in the majority of cases non-adherence is the main reason for therapy failures. However, partnership with the parents and the child is important in order to set individually chosen goals of therapy and may be of help to improve control. Non-pharmacological measures aim at avoiding tobacco smoke, and when a child is sensitised, to avoid allergens. In pharmacological management international guidelines such as the GINA guideline and the British Guideline on the Management of Asthma are leading.

## 1. Introduction

Asthma is a chronic disorder of the bronchial tree, characterized by completely or partially reversible airway obstruction, which may improve spontaneously or may subside only after specific therapy. Airway hyperresponsiveness is defined as the narrowing of the airways as response to a variety of stimuli, such as allergens and nonspecific triggers and infections. Asthma is a chronic disorder of both children and adults, with 300 million individuals afflicted worldwide (Global Initiative for Asthma (GINA) guidelines) [1].

Although the prevalence of asthma has increased over the last decades, especially so in children [2], there is still no sound explanation for this increase. 

Asthma symptoms include recurrent wheezing, coughing, chest tightness, and dyspnea, with nightly and early morning symptoms being more prevalent, whereby quality of life is often reduced [3].

Symptoms of asthma may already occur early in life, with approximately a third of children wheezing during their first three years of life [4]. While the majority of these children will have stopped wheezing by the age of six, 40% will continue to wheeze, having already developed asthma or developing asthma at a later stage in life. Dependent on questioning methodology, up to 10–15% of children may suffer from asthma complaints by school age [5]. In many children, the severity of symptoms diminishes in early puberty and may even disappear altogether, especially in those with mild asthma. However, it is widely known and accepted that symptoms may remain in children with severe asthma or return in early adulthood [6].

Asthma in older children is characterised by a histopathology of a chronic inflammatory process in the conducting airways. Genetic predisposition, in combination with environmental factors, such as allergens and viral infections, may contribute to the development of asthma. Shedding of the epithelial layer is seen, with inflammation and oedema of the airway wall and infiltration of T-lymphocytes, eosinophils, and basophilic cells. This inflammatory process may lead to (or is seen in association with) more structural changes, such as thickening of the basal membrane and hyperplasia of airway smooth muscle and goblet cells, a process commonly known as airway remodelling. Despite observations that lung biopsy specimens from young wheezing children demonstrate the same histopathological pattern [7], little is known about the histopathology in young wheezing children.

Childhood asthma often coexists with allergy and with other atopic diseases. The possible association between allergic sensitization and asthma in children led to the allergic march paradigm. This begins with the development of cow's milk allergy at early age, with symptoms disappearing before the age of 3 years in 95% of the affected children. However, in the years thereafter symptoms occur in other organ systems, resulting in diseases such as allergic asthma, allergic rhinitis, and allergic dermatitis. While approximately 60–75% of school children with asthma have been sensitized to one or more allergens, asthma may also be present without allergic sensitization. It is increasingly accepted that the phenotype of recurrent wheezing, coughing, and chest tightness also occurs in nonallergic individuals. Asthma is therefore considered a heterogeneous disease phenotype with various subphenotypes [8].

While asthma therapy has improved considerably over the last decades, we are still unable to cure the disease. Increased knowledge of possible contributing triggers, and especially the introduction of inhaled corticosteroids during the 1980's, have resulted in better disease control and a reduction in asthma exacerbations. Current medications allow children to live a more or less “normal” life, including participation in sports and other physical and social activities. A small group of children with problematic severe asthma remain the exception. 

This paper focuses on paediatric asthma and its treatment.

## 2. Epidemiology

Although much has been written about the epidemiology of asthma in children, published data are heterogeneous because a uniform definition and uniform methods of data gathering are often lacking. We recently extracted data from PubMed on definitions used to diagnose asthma in paediatric cohort studies (children between 6 and 18 years of age) [9]. Sixty different definitions were seen in 122 papers. The prevalence estimates varied between 15.1% and 51.1%. 

The need for systematic international comparisons of the prevalence of asthma, and the need of a better understanding of different causative and protective factors, led to the International Study of Asthma and Allergies in Childhood (ISAAC) program [10, 11]. The program aimed to elucidate the prevalence in children aged 13-14 years and also in 6-7 year olds. The aim was to initiate an uncomplicated and validated method to measure worldwide prevalence of asthma and allergic diseases. The initial prevalence of self-reported wheezing during the previous 12 months varied from 1.6% to 36.7% in 13- to 14-year-old children from different countries [9]. The corresponding prevalence for parent-reported wheezing in the 6- to 7-year-old children was from 0.8% to 32.1%. Asthma was less prevalent in developing countries, and the highest prevalence was observed in Anglo-Saxon countries. Other conclusions could also be drawn from the study [12]. The authors found an unexpected northwest to southeast gradient in the prevalence of asthma within Europe, and this could not be explained by the recognised risk factors. In addition, asthma prevalence could not simply be explained by genetic differences: significant differences between countries with a similar genetic or ethnic background were seen. Furthermore, there were both differences and similarities in the international pattern of prevalence of asthma, allergic rhinitis, and atopic eczema. The authors found marked differences in prevalence of these three disease entities in the countries with the highest prevalence rates, while the prevalence in the countries with the lowest rates was quite similar. The differences in risk factors and time course of the various disease entities between the different countries could offer an explanation [12].

Additionally, local environmental factors seem to play an important role in the differences in prevalence. Studies of emigrant and immigrant populations, and of Germany after the reunion of East and West, suggest that environmental factors, such as allergens and lifestyle, may explain the observed differences between genetically identical populations [13, 14]. Wang et al. [15] demonstrated that the prevalence of asthma in Chinese adolescents living in Canada and in China differed significantly, despite their common genetic background.

During the last two decades of the previous century, an increase in the incidence and prevalence of asthma was observed in the Western world. In 1989, Strackan proposed a novel, albeit speculative, explanation for this increase of allergic asthma as well as other allergic diseases [16]. He observed that allergic diseases seemed to be prevented by early childhood infections, transmitted by unhygienic contact with older siblings. This explanation entered the world as “the hygiene hypothesis” and led to a maelstrom of studies. However, the increase in incidence and prevalence of allergic disease still remains a mystery to be solved. A cross-sectional study by Shirakawa et al. [17] suggested that tuberculin skin testing in Japanese children reduced the incidence and prevalence of allergic disease. This study seemed to confirm the hypothesis and also suggested that it was possible to skew away from Th2-allergic disease through Th1-inducing infections. However, we found no effect of tuberculin skin testing in a prospective, randomised, double-blind, and placebo controlled study in Dutch children at risk for allergic disease [18]. These conflicting results may be explained by heterogeneous study designs and dissimilar genetic backgrounds, but certainly suggest that the explanation for the increase in allergic disease is not unambiguous. However, at this point the hygiene hypothesis provides the strongest epidemiological explanation for the rise in allergic disease; the probability of asthma is inversely correlated with an increasing diversity of bacterial and fungal taxa in house dust samples, and some viral infections are associated with asthma, while others seem to be protective [19].

Yet, over the last 10 years, a number of studies have suggested that the rising trend in asthma prevalence might have reached a plateau, at least in Australian, Swiss, and Dutch children [21–23]. Possible explanations for this include a true decrease in prevalence, improved identification, and improved environmental influences such as indoor environmental factors, outdoor pollution, and changes in lifestyle, such as a shorter period of breastfeeding.

## 3. Preschool Wheezing

Population studies have shown that one in three preschool children will have at least one episode of wheezing before his or her third birthday, rising to almost one in two (50%) by the age 6 [24, 25]. On the other hand, approximately 80 percent of asthmatic patients start to have symptoms during the first 5 years of life [26]. Recurrent wheezing is frequently reported in preschool children. Usually these symptoms are triggered by the frequently occurring viral upper airway infections. These upper airway infections may occur between six and eight times per year. Martinez et al.'s [25] epic study showed that only forty percent of these initial wheezers continue to wheeze at older age and have, or develop, asthma. 

Unfortunately, ability to predict which children will have *transient* and which will have *persistent *problems is poor. As such, epidemiologic data such as these have limited clinical applicability. In this regard, prospective studies in which subjects were also phenotyped using a number of different clinical measures (e.g., lung function, BAL, etc.), showed considerable overlap between the groups [27]. Therefore, at present, there are no diagnostic tools that can reliably predict the development of asthma among wheezing infants. 

The recognition of wheezing by parents also remains problematic. Noisy breathing is certainly not uncommon among infants. It must be borne in mind that it is difficult for parents to recognize wheezing and the accurate identification of wheezing by medical history is virtually impossible: definitions of terminology used by parents and physicians to describe a variety of symptoms are often quite dissimilar [28]. Children with physician-confirmed wheezing have higher airway resistance than children with parent-reported wheeze [29]. It is not unthinkable that physician-confirmed wheezing may be an important predictor of the development of asthma later. We observed that preschool children with an increased specific IgE, and who also wheezed, had a substantially increased chance of developing asthma by school age [30]. Unfortunately, in this study wheezing was not confirmed by a physician. Devulapalli et al. [31] demonstrated that a high severity score of obstructive airways disease by the age of two, is a strong risk factor for and may predict current asthma at the age of ten. Bronchial biopsies obtained from infants with confirmed wheezing have shown increased thickness of the reticular basal membrane and significantly greater eosinophilic inflammation, as compared to control subjects and even samples from children with parent-reported wheezing [32].

Early identification of asthma is mandatory in school children, since early initiation of treatment in this age group can prevent exacerbations and deterioration of lung function. However, in preschool children data are unavailable. Recent early intervention studies with ICS in young children, aimed at the prevention of asthma, have shown no beneficial results with respect to the development of asthma [33–35], and the results of therapeutic studies are conflicting. An explanation may be that wheezing and coughing at such a young age may be present in a number of different disease entities, with different aetiologies, and it therefore remains difficult to select an effective treatment strategy. 

### 3.1. Phenotypes in Preschool Wheezing

Wheezing disorders in childhood are common and vary widely in clinical presentation and disease course. Various phenotypes have been proposed and classified either by trigger for the wheeze, for example, “episodic viral wheeze” (triggered only by viral colds) or “multiple trigger wheeze” (triggered also by other factors) [36–38]. Phenotypes have also been classified by historical time course, such as “early transient”, “persistent”, or “late onset” [25, 39]. However, these phenotypes do not elucidate whether they represent distinct or different disease entities with separate aetiologies. The three latter phenotypes “early transient,” “persistent,” and “late onset” can only retrospectively be ascertained and are therefore not of clinical or therapeutic relevance. A panel of 7 experienced clinicians from 4 European countries, working in primary, secondary, and tertiary paediatric care, found that preschool wheezing disorders consist of several phenotypes [40]. During structured discussions disease entities could be narrowed to three entities which were linked to proposed mechanisms: (1) allergic wheeze, (2) nonallergic wheeze due to structural airway narrowing, and (3) nonallergic wheeze due to increased immune response to viral infections. Both smoking during pregnancy and prematurity were considered predisposing factors for airway narrowing and therefore should not define separate disease entities. 

In 2008, a task force from the European Respiratory Society defined two phenotypes, “episodic viral wheeze” and “multiple trigger wheeze” (Table 1) [27]. The former is defined as a phenotype where wheezing only occurs during viral colds, while the latter better resembles asthma, with wheezing also occurring without colds and during physical strain, laughing, and so forth. Furthermore, it was advised not to use the term “asthma” in children with preschool wheeze. In addition, up to now there is no prospective validation of the two phenotypes, episodic viral wheeze and multiple trigger wheeze. It may well be that some children with episodic viral wheeze, continue to wheeze and develop asthma, while some children with multiple trigger wheeze stop wheezing by the age of six.

The “allergic wheezing” phenotype, defined by the expert panel, and the “multiple trigger wheezing”, as defined by the ERS task force, are more or less similar. This is also true for the “nonallergic wheezers due to increased immune response to viral infections” and the “episodic viral wheeze.” The most important issue is that the clinical validity of these phenotypes still remains to be prospectively proven. Yet, from a therapeutic perspective, the two phenotypes defined by the ERS task force seem to be the most practical clinical approach at this moment. Other respiratory tract diseases that cause wheezing, such as gastroesophageal reflux, anatomic abnormalities of the airways, aspiration of foreign bodies, immune deficiencies, cardiac abnormalities, and cystic fibrosis, should be excluded.

### 3.2. Predicting Asthma amongst Wheezing Preschool Children

Periods of viral-induced wheezing, cough, and chest tightness occur in many children and currently it remains difficult, if not impossible, to identify which child is at risk of developing asthma later in life. In order to effectively treat the preschooler with asthma, it is necessary to identify the asthma early in the course of the disease. Yet, in order to avoid overtreatment, including the possible side effects of especially ICS therapy, the child with transient wheezing episodes also needs to be identified early.

Respiratory syncytial virus (RSV) and rhinovirus (RV) have both been linked to initial wheezing episodes and to the risk of recurrent wheezing in early childhood [41]. RSV infections during the autumn and winter are of major importance for clinicians that, due to the many emergency room consultations and clinical admissions, they necessitate. Yet, RV infections during the first three years of life have a significantly stronger association with the development of asthma, by the age of six years, than RSV infections in early life. When considering wheezing during the first three years of life, Jackson et al. [41] showed that wheezing with RSV alone was associated with an increased risk (OR 2.6) of asthma by the age of 6, compared with children who did not wheeze with RV or RSV. Wheezing with RV, regardless of RSV wheezing history, was associated with an even greater increased risk of asthma by the age of six (OR 10.0). 

However, children with early-onset allergic sensitisation and recurrent respiratory wheezing seem to be at risk for asthma in adolescence and adulthood. These observations suggest that both respiratory viral infections and allergic sensitisation may injure the airways, resulting in reversible airway narrowing and bronchial hyperresponsiveness to external stimuli, and may lead to continued wheezing. 

Various predictive models of clinical indicators of risk have been proposed. Castro-Rodríguez et al. [42] proposed the Asthma Predictive Index (API) in order to assess which child would continue to wheeze at school age (Table 2). They formulated a parental history of asthma and physician diagnosed atopic dermatitis as major criteria, and physician diagnosed allergic rhinitis, wheezing unrelated to colds, and blood eosinophils ≥4% as minor criteria. Using the API, a child with recurrent wheezing and 1 major criterion or 2 of 3 minor criteria had a 4.3 to 9.8 times greater risk of asthma at school age. A few years later the API was modified because allergic rhinitis is difficult to diagnose in young children [20]. Allergic sensitization to ≥1 aeroallergen was added as major criterion, while physician diagnosed allergic rhinitis, allergic sensitization to milk, egg, or peanuts were added as a substitute for the minor criterion.

Several other predictive models have subsequently been proposed [31, 43–46], yet few (the API, the modified API, and the PIAMA risk score) [20, 42, 45] have been externally validated. In a recent evaluation of the predictive performance of the API and the modified API in the Tucson group, Leonardi et al. [47] also validated both forms of the API in the 1998 Leicester cohort [48]. Using the API and simpler prediction rules, based only on wheeze frequency and a random rule, to elucidate the predictive performance of chance, they attempted to predict asthma at school age. The predictive performance of the API in the Leicester cohort was, although comparable to the original study, modest and similar to the prediction based only on the frequency of preschool wheeze [47]. Savenije et al. recently published a review of this subject [49]. Similar to the results of Leonardi et al., they concluded that the currently available prediction rules, aiming to identify preschool children having asthma at school age, are of modest clinical relevance. The authors suggested that the prediction rules used may be enhanced by a more precise definition of risk factors, by the addition of exposures and interaction with exposures with other risk factors, by more precise phenotyping with objective measures, by the combination of noninvasive techniques with developed prediction rules, and by the addition of a personal genomic risk profile. Although the conclusions drawn are valid, the use of this combination is currently not feasible or relevant in daily clinical practice.

## 4. Childhood Asthma

It is easier to diagnose asthma in school-aged children (6 years and older) than in the preschoolers presenting with recurrent wheezing, coughing, chest tightness, and breathlessness, where the idiom “not all that wheezes is asthma” is applicable. For the younger children, the diagnosis is usually suspected based on a typical history of recurrent wheeze, cough, chest tightness, and breathlessness. These symptoms are certainly not pathognomic for asthma. However, most other respiratory diseases presenting with these same recurrent symptoms are rare (Table 3), and it is common practice to commence asthma therapy without first excluding these other respiratory conditions.

Approximately 60–75% of school-aged children with asthma have an allergy [50]. Until the age of 14 years, the incidence and prevalence of asthma is higher in boys than in girls. Paradoxically, during puberty the ratio seems to change, resulting in a higher incidence in girls than in boys [51, 52]. Moreover, the number of remissions in boys is greater than in girls, and young females tend to have more severe asthma [53]. The latter may be influenced by female hormones since early menarche is associated with a decline in lung function [54].

### 4.1. The Burden of Childhood Asthma

Childhood asthma is common in the Western world and underdiagnosed in minority populations in Europe and the United states. Minority populations are significantly burdened by asthma morbidity [55, 56] and suffer higher rates of emergency department visits, hospitalization, and even death [55]. Quality of life (QoL) in childhood asthma is affected by asthma control. The better the asthma control, the better the QoL is. Uncontrolled asthma is associated with a reduced lung function, impaired performance in physical exercise, and impaired QoL [57].

Most asthma symptoms occur at night. Almost half of asthmatic children presenting at a university hospital outpatient clinic suffered from nocturnal symptoms [58]. Nocturnal symptoms cause loss of sleep [59]. Even in children with stable asthma, quality of sleep is diminished [60]. Sleep disruption influences daily activities, such as school attendance and performance. Nocturnal awakening may also cause parental work absenteeism, and may disrupt family life [61]. More severe asthma leads to more frequent school absenteeism which may negatively affect an individual's level of education and, possibly, choice of career. Furthermore, frequent nocturnal awakenings may cause depression, aggressive behaviour, and attention problems in adulthood [62].

Exercise, induced airway obstruction (EIAO) is yet another burden for children with asthma. Together with the frequent nocturnal awakenings due to dyspnea, EIAO may hamper social contacts. Exercise is a common trigger of bronchial hyperresponsiveness and may cause cough, wheezing, and chest tightness [63, 64]. EIAO is indicative of insufficiently controlled asthma [65].EIAO occurs in up to 23% of school children and has serious repercussions on the quality of life of these children [66, 67]. EIAO limits participation in sports and play, and 79% of children experience EIAO as the most burdensome of their asthma [68, 69]. EIAO is highly specific for childhood asthma, as it is indicative of airway inflammation [66, 70]. Sports and play are of great importance for a child, stimulating the development of both social and motor skills. Unfortunately, symptomatic history is neither a sensitive nor a specific tool for diagnosing EIAO.[71–73] EIAO may induce reluctance to exercise and a sedentary lifestyle, which in turn may lead to a low cardiovascular fitness and an increased body mass index (BMI). Lower cardiovascular fitness results in higher breathing rate at a relatively low work load, which is the trigger for EIAO. Furthermore, an increased breathing rate results in a feeling of dyspnea, which may be misinterpreted by children and their parents as EIAO. An increased BMI has been associated with bronchial hyperresponsiveness for both exercise and methacholine [71, 74–76]. In conclusion, asthmatic children with a low cardiovascular fitness and/or a high BMI, compared to peers, will have a relatively higher breathing rate during play and sports. This, in turn, increases the trigger for EIAO, further compromising athletic performance and QoL.

Childhood socioeconomic status in the United States seems to be strongly associated with the onset of chronic diseases such as asthma. In a longitudinal population based study in the USA, paternal education was negatively associated with the risk of developing asthma. Maternal education was correlated to high school dropout [77]. Hatzmann et al. studied the QoL consequences in parents of Dutch children with various chronic diseases. The largest subgroup was parents of children with asthma. Parents of all groups had a significantly lower health-related quality of life. Subgroup analysis showed lower health-related quality of life with respect to sleep, social functioning, daily activities, vitality, positive emotions, and depressive emotions in disease-specific groups [78].

### 4.2. Asthma Control

The goal of asthma therapy in children is to achieve asthma control by optimizing lung function, reducing day and night time symptoms, reducing limitations in daytime activities and the need for reliever treatment, and by reducing asthma exacerbations [79]. However, especially in children, it is important to achieve control with a minimum of medication side effects.

Asthma control is assessed by the presence of daytime symptoms, limitation in activities, nocturnal symptoms and awakenings, need for reliever medication, and lung function assessment in children from the age of 6 (Table 4). Total control is possible, but optimal effect of medication is often hampered by poor adherence and poor inhalation technique. Apart from these factors, additional conditions such as dysfunctional breathing, allergic rhinitis, obesity, and mental condition may hinder optimal control. Clinical practice shows that many children are not well controlled [80, 81]. In the latter (Swiss) study, asthma control was excellent in only 18% of children [81], satisfactory in 33%, and unsatisfactory with disrupted sleep, restricted activities, and school absence in 49%. In addition, the authors found a mismatch between poor asthma control and perception of control by the parents. Eighty-nine percent of the parents of children with poor asthma control were actually satisfied with the results of treatment. The same misconception applies for physicians. Van den Berg et al. [82] interviewed 118 Dutch paediatricians and 152 general practitioners, in order to assess the view of the physician with respect to the patient's asthma management. A questionnaire was used with similar questions to those of the AIRE study [83]. Dutch physicians believed that the asthma in the majority of their patients is well controlled and underestimate the prevalence of daytime symptoms. They believe that their patients are aware of the differences between reliever medication and maintenance medication and overestimate the number of patients in possession of a written action plan.

Asthma control in the GINA guidelines aims at improving control by assigning each patient to one of five treatment steps [1]. If a patient is not well controlled, depending on the medication step he or she started with, controller medication should be started, or the dose should be increased. In adult patients, the Goal study investigated this strategy [84]. A 1-year, randomized, stratified, double-blind, and parallel-group study of 3421 patients with uncontrolled asthma compared fluticasone and salmeterol/fluticasone in achieving totally and well-controlled asthma. Treatment was stepped up until total control was achieved (or maximum 500 *μ*g corticosteroid twice a day).Only 19% of the adult patients on fluticasone and 41% of on salmeterol/fluticasone achieved total control.

This study emphasizes that other, previously mentioned, factors such as poor adherence to medication, poor inhalation technique, and unrecognised comorbidity play an important role. Physicians and nurses should recognise and try to improve these behavioural aspects. Seeking partnership with parents and children and setting individual chosen goals of therapy may be helpful in improving disease control. A written self-management plan and asthma education aimed at better perception by parents and children may enhance success [85–87].

### 4.3. Problematic Severe Asthma

The majority of children with asthma are easy to manage with occasional bronchodilator use or a low or moderate dose of ICS. Children who are referred to specialist care, but do not respond to standard therapy, are defined as having problematic severe asthma. The burden for this group is severe. Children with uncontrolled asthma despite high dose ICS and controller therapy have a decreased QoL, consume a disproportionate amount of resources, and may die prematurely [88]. The exact prevalence of this group is hard to estimate but is probably approximately 5% of all children with asthma or 0.5% of the pediatric population [89]. The key question to pose is whether these children have been sufficiently evaluated over a period of time by a specialist [90]. A number of studies have shown that the majority of these children ultimately receive a different diagnosis have poor adherence to their medication or have a poor inhaler technique [91, 92].

Problematic severe asthma needs careful evaluation. The patients form a heterogeneous group requiring a specific workup. The group consists of (1) children with an incorrect diagnosis of asthma; (2) children with asthma in addition to another disease; (3) children with difficult asthma (which can be improved after optimizing basic management) (4) children with therapy resistant asthma (severe symptoms despite the implementation of all the basic management steps).

### 4.4. Incorrect Diagnosis of Asthma

Asthma may be mimicked by other diseases such as dysfunctional breathing (hyperventilation or vocal cord dysfunction, VCD), malformations of the airway anatomy such as a tracheal malacia, or vascular anomalies such as a vascular ring. Other diseases that should be excluded are cardiac anomalies, immune deficiencies, primary cilliairy dyskinesia, cystic fibrosis, bronchiectasis, obliterative bronchiolitis, inhaled foreign body, allergic rhinitis and gastroesophageal reflux.

### 4.5. Asthma plus Another Disease

Asthma itself may be mild or moderate, but comorbidities such as those mentioned above may also be present. VCD is often seen in conjunction with asthma and is a respiratory condition characterized by adduction of the vocal cords, resulting in airflow limitation at laryngeal level. Newman et al. [93] found that 53% of laryngoscopically confirmed adult VCD patients also had asthma. Most of the patients were young woman and with an average of 4.8 years of misdiagnosed asthma. Typically, the abnormal breathing sounds disappear when the child with VCD is asleep, and in contrast to asthma, a child with VCD does not suffer from nocturnal awakenings. Other conditions, as mentioned under Incorrect Diagnosis of Asthma, as well as psychosocial factors, should be considered as co-morbidities. 

### 4.6. Difficult Asthma

Difficult asthma is defined as that which is poorly controlled despite a daily dose of at least 800 *µ*g budesonide or equivalent for a minimum of six months [94]. Symptoms may be worsened by continuing environmental factors, such as smoking by the parents or the child, or allergens [95]. Environmental causes of secondary steroid resistance, such as continuing allergen exposure or environmental tobacco smoke exposure, should be identified [96–98]. 

It is therefore necessary to evaluate the home situation, and to contact the general practitioner and school. In a multidisciplinary consultation with physicians and asthma nurses, all results of the history, adherence to therapy, inhalation technique, allergy testing, lung function measurements (spirometry and bronchial hyperresponsiveness challenges), the results of the home visit for the evaluation of environmental triggers, and psychosocial issues need to be addressed [90].

It is worthwhile attempting the reduction in symptom levels in difficult asthma. Not only because of current symptoms, but also to reduce future risks [90], such as failure of normal lung growth [99], risk of loss of future asthma control, risk of future exacerbations, risk of long-term chronic obstructive pulmonary disease (COPD) [100], and risk of medication side effects.

### 4.7. Therapy Resistant Asthma

Once all has been checked, what remains is classified as severe therapy resistant asthma. Bush and Saglani proposed a stringent workup of these patients [101]. The child should be assessed prior to, and after, a steroid trial with injection of triamcinolon. Assessment should include airway symptoms (asthma control test), use of rescue medication, lung function (spirometry, reversibility after administration of a short-acting *β*_2_ mimetic), airway inflammation (exhaled nitric oxide, induced sputum, and fibreoptic bronchoscopy with bronchoalveolar lavage and endobronchial biopsy). 

With this protocol Bush and Saglani attempt to answer the following questions: (1) what is the pattern of airway inflammation, (2) is there concordance between symptoms and inflammation, (3) does the child have steroid responsive asthma, and (4) does the child have persistent airflow limitation? This protocol may enable physicians to better phenotype this small group of children and may help to better future therapies.

## 5. Treatment of Wheezing Preschool Children and of School Children with Asthma

### 5.1. Nonpharmacological Measures

A number of nonpharmacological measures to improve symptoms and disease outcome should be discussed with the parents. As previously mentioned, partnership with parents is important in order to set individually chosen goals of therapy and may be of help in improving control of the disease. Furthermore, possible fear of side effects of drugs should be discussed. Inhalation technique should be trained, and a written self-management plan should repeatedly be discussed on several occasions. Nonpharmacological measures are similar in preschool children and children above the age of 6. However, there are also nuances with respect to children in puberty. The asthma team should be aware that children may start to smoke before the age of 15, mostly because of peer group pressure [102]. Another point of concern is (lack of) adherence to medication.

### 5.2. Tobacco Smoke

Environmental tobacco smoke induces wheezing in the preschool child. Exposure to environmental tobacco smoke *in utero* is strongly associated with preschool wheezing [4, 103]. After birth it may also induce wheezing and lead to exacerbations [104]. Moreover, children exposed to smoke are more likely to smoke later in life and are prone to develop COPD [105, 106].

### 5.3. Allergen Exposure

There is no evidence that avoidance of allergens improves symptoms in children with viral wheeze. In the case of clinical evidence of allergy and allergic sensitisation, symptoms may be aggravated by exposure. Various population studies have demonstrated that children growing up with pets are less likely to develop sensitisation to pets [107, 108]. This may be due to selection bias since families with allergic individuals tend to avoid having pets. 

House dust mite (HDM) reduction measures have been shown to reduce HDM levels in households [109] but have not been shown to improve asthma symptoms in children [110].

### 5.4. Pharmacological Management

The pathogenesis of recurrent wheezing, coughing, chest tightness, and breathlessness at preschool age is heterogeneous. This is an explanation for the variation in effectiveness of the different drug therapies in the younger age group. Low drug deposition in the lungs despite optimal inhalation, anatomy of the upper airways, and difficulty in inhaling medications in a correct manner may explain the moderate effectiveness of the different medications in preschool children [111]. Lung deposition may be improved by using a pressurized metered dose inhaler (pMDI) with extra-fine particles. However, even if the most optimal device is chosen, correct administration remains the single most important determinant for efficient drug delivery. A small facemask leak may dramatically reduce the delivered dose, making a good seal essential. The dosage reaching the lungs is minimal during crying [112, 113].

Worldwide, different guidelines for the management of asthma in childhood are in use. Many countries have a unique guideline. The GINA guideline and the British Guideline on the Management of Asthma are leading and available for many physicians around the world [1, 114].  Table 5 shows pharmacological management in preschool wheezers of 5 years and younger as suggested in the GINA guidelines [79].

### 5.5. Bronchodilators

#### 5.5.1. Short-Acting *β*_2_Agonists

Inhaled short-acting *β*_2_ agonists are the drug of choice as short-term rescue therapy. Inhalation leads to low systemic exposure and therefore reduced side effects. All wheezing children (preschool wheeze and schoolchildren with asthma) should be treated with short acting *β*_2_ agonists, on an “as needed basis.” The 2012 revised edition of the British Guideline on the Management of Asthma shows that the evidence of efficacy of these drugs in the very young (< 2 years) is low, and studies are controversial in especially this age group [114]. Nevertheless it has been demonstrated that these drugs are able to bronchodilate [115, 116]. Paradoxical reactions have also been described in the very young [117].

In older children, there is strong evidence that short-acting *β*_2_ agonists are effective. There seems to be no difference in efficacy between regular and “as needed” use [118].

#### 5.5.2. Long-Acting *β*_2_Agonists

Long-acting *β*_2_ agonists (LABA) are not recommended in the age group of 5 years and younger, by both the GINA guideline and the British Guideline on the Management of Asthma. There are no randomised controlled trials in this age group and insufficient safety data [79, 114].

In school-aged children with asthma, LABAs are effective, but less effective than in adult patients with asthma. In an earlier double-blind randomized controlled trial in asthmatic schoolchildren aged 7–15 years, children were randomized to salmeterol 50 *μ*g twice daily or placebo twice daily. All children were already on treatment with an inhaled corticosteroid. After a followup of 16 weeks, we found a small but significant effect in FEV_1_ in favor of the salmeterol group (difference between groups 4.9 ± 2.0% predicted) but no effect on the degree of bronchial hyperresponsiveness [119]. In a randomized, controlled, and three-armed parallel study in children aged 6–16 years, comparing 200 *μ*g beclomethasone diphosphate (BDP) twice daily with 400 *μ*g BDP twice daily and 200 *μ*g BDP + 50 *μ*g salmeterol twice daily, no differences were seen between the three arms after a followup of 52 weeks, with respect to symptoms, FEV_1_ and the degree of bronchial hyperresponsiveness [120]. More recently a double-blind, multicentre, randomized, controlled trial was performed in children aged 6–16 years with symptomatic asthma. Salmeterol/fluticasone propionate 50/100 *μ*g twice daily was not inferior to fluticasone propionate 200 *μ*g twice daily, with regards to efficacy of symptom control and lung function, which was similar in both groups [121]. A Cochrane systematic review of the addition of salmeterol to controller medication with an ICS concurred with the conclusions of these studies, that there is only a limited effect of LABA in school children with asthma. The conclusion of this systematic review is that addition of LABA to an ICS provides no reduction in exacerbations, no improvement in QoL, and no reduction in short acting *β*_2_ agonist usage. Only a small (80 mL), albeit significant improvement in FEV_1_ was found [122].

In preparation for the December 2008 Advisory Committee, the Food and Drug Administration (FDA) conducted a meta-analysis of 110 studies evaluating the use of LABAs in 60954 patients (adults and children) with asthma (http://www.fda.gov/Drugs/ResourcesForYou/HealthProfes-sionals/ucm219161.htm). The meta-analysis used a composite endpoint to measure severe exacerbation of asthma symptoms (asthma-related death, intubation, and hospitalization). The results of the meta-analysis suggested an increased risk for severe exacerbation of asthma symptoms in patients using LABAs compared to those not using LABAs. The largest risk difference per 1000 treated patients was seen in children 4–11 years of age. The results of the meta-analysis were primarily driven by asthma-related hospitalizations. Based on these findings, the FDA decided that LABAs should only be used as additional therapy for patients with asthma who are currently using ICS, but are not adequately controlled. LABAs should not be used in patients whose asthma is adequately controlled on low or medium dose ICS. Use of a LABA alone without use of an ICS is contraindicated.

### 5.6. Control Medication

#### 5.6.1. Inhaled Corticosteroids

The results of therapeutic studies are conflicting. In many preschool children, wheezing occurs in association with colds. The majority of these children have “viral wheeze,” whereby the wheezing episodes coincide with viral colds. As a generalisation, these children have no clinical signs of allergic disease nor are they sensitized to allergens. These children wheeze intermittently and are symptom-free between episodes. The efficacy of ICS treatment for episodic viral wheeze in preschool children is controversial. The majority of asthma exacerbations in school-aged children are associated with viral infections [123], and this is also true for the majority of wheezing episodes in preschool children [124]. Intermittent versus daily ICS treatment in children was reviewed by the Cochrane Airways Group [125]. Studies in children up to 17 years of age were included, but the review also contained studies conducted in preschool children. This paper showed that children benefited from intermittent use of high-dose ICS (1600–3200 *μ*g/day BDP or BUD) as evidenced by a reduction in the severity of symptoms. There was also a trend towards reduced necessity of oral corticosteroids. More recently, a controlled, randomised, and double-blind clinical trial of 750 *μ*g FP versus placebo twice daily in 129 children aged 1–6 years, with recurrent virus-induced wheezing, showed a reduction in the use of rescue oral corticosteroids in the FP-treated patients [126]. However, treatment with FP was associated with a reduced height and weight gain. Among preschool children, no benefit was seen after continuous low-dose ICS treatment (400 *μ*g/day BUD), with respect to a reduction in the number or severity of wheezing episodes [127]. Finally, intermittent 2-week courses of inhaled budesonide (400 ug/day), during wheezing episodes, showed no benefit during the first three years of life, in a double-blind, placebo controlled, and randomised interventional study [128].

Maintenance treatment with low-to-medium dosage ICS for episodic viral wheeze seems to offer no benefit. Intermittent treatment with high-dose ICS during wheezing episodes has some beneficial effect but increases the risk of systemic side effects. An alternative possibility for this phenotype is treatment with montelukast, which reduced the rate of wheezing episodes by 32%, in comparison to placebo, in 549 preschool children with episodic viral wheeze [129].

Wheezing preschool children with an allergic phenotype, such as children with allergic dermatitis or with allergic sensitisation, are associated with allergic asthma at school age and adolescence. In these pre-schoolers, viral infections are also often the trigger for wheezing and coughing. Yet, the children also wheeze between colds. Symptoms may also be triggered by crying, laughter, and physical effort. Treating preschool children with multiple-trigger wheeze and with ICS appears to be more successful than that of children with episodic viral wheeze. Based on these findings, many believe that multiple-trigger wheeze resembles allergic asthma, but there is little direct evidence to support this. It remains unclear whether the histopathology of the airways from children with multiple-trigger wheezing resembles that of allergic asthma. However, a proportion of preschoolers with persistent wheeze do develop asthma in later life [4, 130].

Literature reviews of the efficacy of ICS in recurrent wheezing preschool children [48, 131] and a number of randomised, double-blind, and placebo-controlled clinical trials, conclude that continuous treatment with ICS decreases the number of days with symptoms among children with persistent wheezing. It does not reduce the frequency of hospitalisation [132] but does reduce wheezing/asthma exacerbations and leads to improved symptoms and lung function, respectively [133].

There is solid evidence that maintenance treatment with a low-to-moderate dose of ICS decreases the number of days with asthma symptoms, in children with multiple trigger wheeze. However, the question of whether the relative benefit of continuous treatment with ICS (approximately 5% fewer symptom-free days versus placebo) is clinically significant and outweighs the possible side effects remains pertinent [131].

Small-particle ICS, such as ultrafine HFA-BDP aerosol (QVAR) and ciclesonide may offer a potential benefit in preschool children. This resulted in a recommendation in the 2007 revised Dutch Paediatric Asthma Guidelines that children under six years of age be treated with a small particle ICS [134]. Ironically, the efficacy of small particle-inhaled corticosteroids in preschool children has not yet been evaluated in prospective clinical trials. For this reason HFA-BDP in the Netherlands is registered from the age of five years and older in contrast to the recommendation in the 2007 revised Dutch Paediatric Asthma Guidelines. The only study that suggests that small-particle ICS may be advantageous in very young children is an infant model study. In an anatomically correct model of the upper airway of a 9-month-old infant, the SAINT model [135], lung deposition of CFC BDP (MMAD 3.5–4.0 *μ*m), and ultrafine HFA-BDP (MMAD 1.1 *μ*m) were compared. The SAINT model was connected to a breathing simulator and a cascade impactor. This study showed that lung deposition of ultrafine HFA-BDP was 25.4%–30.7% over the range of tidal volumes evaluated (50 mL–200 mL), while the lung deposition for CFC BDP ranged from 6.8% to 2.1% [136]. The deposition of the small particles was relatively independent of tidal volume, which theoretically, may be an advantage in young children. This study suggests that ultrafine HFA-BDP offers a better lung dosage in preschool children compared with an ICS with a larger MMAD. However, these data must be interpreted with the caveat that drug delivery for individual patients in clinical practice also depends on other factors, such as inhalation technique and cooperation of the child. 

Directly after their introduction in the eighties of the former century, ICS became the cornerstone of asthma therapy in school aged children and adolescents (Table 6). In Europe, ICS has completely replaced sodium cromoglycate in the market place because of better efficacy. For this reason sodium cromoglycate has had no place in European guidelines for a number of years. It was convincingly demonstrated that ICS treatment of the underlying airway inflammatory processes provided overall asthma control that was far superior to bronchodilator treatment alone. In children aged 7–16 yrs, Van Essen-Zandvliet et al. [137] showed that the effects of chronic treatment with BUD (100 *μ*g administered 3 times per day for 22 months) was far superior to chronic treatment with the short acting *β*_2_ adrenergic drug salbutamol (200 *μ*g administered three times per day), with respect to asthma symptoms, lung function, degree of BHR, and frequency of exacerbations. Eight years later, these results were confirmed in a much larger population of school children with mild-to-moderate persistent asthma of approximately the same age group (5–12 yrs) in the USA, receiving budesonide 200 *μ*g, nedocromil 8 mg, or placebo twice daily for 4–6 years [138]. The results of these and other paediatric asthma studies provide a solid foundation for our current understanding of ICS and their role in the treatment of childhood asthma. It is safe to conclude that inhaled corticosteroid treatment is very effective in school aged children.

#### 5.6.2. Montelukast

Montelukast is a leukotriene receptor agonist that is approved for the treatment of preschool children older than 5 months of age. It is provided as granules or chewable tablets, both of 4 mg for children aged between 5 months and 5 years and 5 mg chewable tablets for children between 6 and 14 years of age. 

In 3- to 5-year-old preschool wheezing children, montelukast provided bronchoprotection against provocation with cold air [139]. A few years later, montelukast was confirmed to indeed reduce the degree bronchial hyperresponsiveness in preschool children [140]. Montelukast improved symptoms and achieved a 30% reduction in exacerbations in 689 preschool children with multiple-trigger wheeze [50]. Montelukast also reduced the rate of wheezing episodes by 32% compared to placebo in 549 preschool children with episodic viral wheeze [129]. These earlier studies indicate that montelukast is a good alternative for ICS if treatment is not successful or when inhalation of ICS is not possible due to lack of cooperation by the child. In a comparison with nebulised budesonide inhalation suspension in children aged 2–8 years, no difference was observed between montelukast and budesonide, with respect to the primary outcome, time to first additional asthma medication at 52 weeks [141]. Time to first additional asthma medication was longer at 12 weeks, and exacerbation rates were lower over a period of 52 weeks for budesonide versus montelukast. Time to first severe exacerbation (requiring oral corticosteroids) was similar in both groups, but the percentage of subjects requiring oral corticosteroids over a period of 52 weeks was lower with budesonide (25.5% versus 32.0%). Peak flow and Caregiver and Physician Global Assessments favour budesonide [141]. The results in this study are in favour of budesonide and in line with other studies in school aged children. However, a direct comparative study between montelukast and an ICS, in preschool children, is not available in a literature search [27, 51].

 Another possibility is to only treat the wheezing preschooler during wheezing episodes. Robertson et al. [142] started periodic treatment with montelukast at the beginning of a cold. This significantly reduced unscheduled healthcare visits but had no effect on hospitalisation, duration of the wheezing episodes, or oral courses of steroids.

Montelukast is an additional therapeutic option in school aged children with asthma. In a double-blind, placebo controlled trial, 6–14-year-old children with asthma received either montelukast (5 mg chewable tablet) or placebo once daily at bedtime for 8 weeks. The study showed that montelukast improved morning FEV_1_ compared to placebo [143].

Montelukast seems less effective than ICS in school children with asthma. In a 12-month, multicenter, randomized, double-blind, and noninferiority trial in children aged 6–14 years, the effect of once-daily 5 mg montelukast was compared to inhaled fluticasone (100 *μ*g) twice a day. The primary outcome variable was the percentage of asthma rescue-free days [144]. Montelukast appeared not to be inferior to fluticasone in increasing the percentage of rescue-free days in these school children with mild asthma. However, secondary endpoints, including FEV_1_ % pred, days with beta-receptor agonist use, and QoL, improved in both groups, but were significantly better in the fluticasone treatment group. These observations are in line with a study in 6–17-year-old children with mild-to-moderate asthma, where the authors sought to determine intraindividual and interindividual response profiles and predictors of response to an ICS and montelukast [145]. They found improvement in most asthma control measures for both controllers. However, the Asthma Control Questionnaire scores, spirometric values, and inflammatory biomarkers (exhaled nitric oxide, eNO) improved significantly more with fluticasone than with montelukast therapy. In a randomized, double-blind parallel study, three treatment regimes were compared over a treatment period of 48 weeks [146]. The treatment regimens were fluticasone 100 *μ*g twice a day, fluticasone 100 *μ*g/salmeterol 50 *μ*g in the morning and salmeterol 50 *μ*g in the evening, and montelukast 5 mg in the evening. Both fluticasone monotherapy and the fluticasone/salmeterol combination achieved greater improvements in asthma control days than montelukast. However, fluticasone monotherapy was superior to the fluticasone/salmeterol combination in achieving other dimensions of asthma control. Fluticasone monotherapy was superior to montelukast for asthma control days and for all other control outcomes (FEV_1_, maximum bronchodilator response, bronchial hyperresponsiveness, and eNO). 

#### 5.6.3. Other Control Medication

The GINA guidelines advocate other possibilities for controller medication [1]. Sustained release theophylline in steps 3 and 4 as treatment options and anti-IgE antibody (omalizumab) in step 5. Theophyllines have long been known as bronchodilators and low dose therapy may have anti-inflammatory properties. The addition of low-dose theophylline to moderate-dose ICS, in asthmatic adults, is more effective in patients with severe asthma than increasing the dose of ICS to the maximum tolerated dose [147]. Subsequent withdrawal of theophylline causes a loss of asthma control in adult patients with severe asthma [148]. In smoking adult patients who became refractory to steroids, theophylline is effective when added to a dose of ICS, shown to be ineffective as monotherapy [149]. However, most European guidelines do not advise the use of theophylline because of the toxic side effects and drug interactions.

Omalizumab is an expensive therapeutic option and has the inconvenience that it needs to be administered subcutaneously in hospital, under supervision. It is a therapeutic option in step 5 for therapy resistant asthma, after the exclusion of all other possibilities, following the stringent work-up for this specific group as mentioned above [101]. Omalizumab has been shown to reduce exacerbations, reduce the dosage of inhaled (and oral) steroids, and improve asthma related QoL [150, 151]. Moreover, safety has been demonstrated in studies with 1-year duration [152]. The therapy is recommended in children with atopic asthma ≥ 6 years, with an upper IgE limit of 1300 IU. Yet, substantial numbers of children have higher levels [153].

## 6. Guidelines

Guidelines for asthma diagnosis and treatment have been in use for at least two decades. Most countries in the Western world have their own guidelines or use an internationally accepted guideline such as the GINA guideline or the British Guideline on the Management of Asthma [1, 114]. The latter guideline has the advantage that the level of evidence for each step is mentioned. A portion of the standard medication steps are based on little or no evidence. This may be due to the lack of efficacy of drugs, such as in preschool children, where it is impossible to predict the phenotype, benefitting from a certain drug. Another reason is a lack of studies that support decision making. The latter is the case in steps 4 and 5 of the GINA guideline. A single study supports the choice to step up low-dose ICS therapy in a child with uncontrolled asthma [154]. In this study, 182 children, aged 6 to 17 years, who had uncontrolled asthma, despite receiving 100 *μ*g of fluticasone twice daily, were randomized to three treatment arms. The arms included 250 *μ*g of fluticasone twice daily (ICS step-up), 100 *μ*g of fluticasone plus 50 *μ*g salmeterol (LABA step-up) twice daily, or 100 *μ*g of fluticasone twice daily plus 10 or 5 mg montelukast. The dosage montelukast depended on the age of the child. Study follow-up time was 16 weeks. The authors, using a triple-cross-over design, compared three outcomes (exacerbations, asthma control days, and the FEV_1_) to determine whether the frequency of the differential response to the step-up regimes was more than 25%. They observed clinically significant improvement in almost all children. The response to LABA step-up was the best response, as compared to the montelukast response and ICS response. However, many children had a best response to montelukast or ICS step-up. This indicates that one may have to test which drug of choice is needed in the individual patient, if step-up is from low to moderate dose of ICS, it is required in uncontrolled asthma. Most children appear to benefit from the addition of LABA, but many children benefit from doubling the dose of ICS and benefit from the addition of montelukast.

Adherence to guidelines, by health care professionals, remains suboptimal. In 18 Primary Health Care Centres in Stockholm, Sweden, medical records from 424 children with asthma were selected and investigated [155]. The medical records were searched for documentation of the most important indicators of quality, as stipulated in the Swedish national guideline, that is, tobacco smoke, spirometry, pharmacological treatment, patient education, and inhalation technique. Only 22% of the children aged 6 years and older had performed spirometry. Fifty-eight percent of the children used ICS on a daily basis, but documented education and demonstration of inhalation technique was only present in 14% of the charts, and exposure to tobacco smoke was documented in 14% of the children. The authors conclude that the adherence by healthcare professionals to guidelines for asthma is poor and that there is considerable room for improvement. The implementation of pharmacological treatment appeared to be better than nonpharmacological measures, such as documented education, demonstration of inhalation technique. In this study the presence of an asthma nurse was not associated with improvement of most non-pharmacological measures [155]. Patients who reported having visited the asthma nurse during the previous year, had more knowledge but similar asthma control and quality of life, compared to patients who reported no visit. Spirometry was more readily performed in children consulting the asthma nurse. We showed, in a three-armed randomised open study, that the quality of care provided by an asthma nurse was similar, compared to care given by a paediatrician or general practitioner [156]. In contrast to other studies, we showed that access to an asthma nurse improves asthma control, knowledge, and QoL [157, 158]. In a recent Canadian study on adherence to paediatric asthma guidelines in an emergency department, the authors collected information from healthcare professionals regarding their knowledge, attitudes, and use of a care pathway, for acute childhood asthma [159]. Fifty-six percent of the healthcare professionals responded, and 99% of the responding professionals were familiar with the pathway, 90% agreed with its use for mild and moderate asthma, while 79% agreed with its use in severe asthma. A majority (64%) admitted to deviating from the pathway. The authors concluded that the majority of healthcare professionals had a positive attitude toward the pathway. Knowledge gaps and the balance between standardization and individualization of care were thought to be key elements in explaining suboptimal adherence. However, despite the positive attitude towards the pathway, both studies show suboptimal adherence to guidelines [155, 159]. This is in agreement with many clinical practices. Suboptimal adherence to guidelines is generally the result of a lack of implementation strategies after publication of the guideline. It is well known that it is difficult to introduce (new) evidence and guidelines into routine clinical practice. In an overview, Grol and Grimshaw discuss different approaches for transferring evidence into practice [160]. In their overview, they state that plans for change should be based on characteristics of the evidence or guideline itself, barriers and facilitators of change. Changes in clinical practice are only partly within the control of physicians. The patient, the organisation of care processes, resources, leadership, and political environment also play an important role [161]. Grol and Grimshaw advise interactive and continuous education, including discussion of evidence, local consensus, feedback on performance, and making personal and group learning plans. [160].

## 7. Conclusion

Asthma is one of the most chronic disorders in children. The prevalence of asthma has increased during the last decades but seems to have reached a plateau. The burden of asthma is considerable. It influences quality of life, may prevent children from participating in sports and play, may hamper social contacts, and may cause school absence and hamper career development. Asthma begins in early life. Before the age of 6 many children wheeze, but only 40% of these early wheezers develop asthma. There appear to be different phenotypes. A task force from the European Respiratory Society (ERS) proposed to use two different phenotypes: “episodic viral wheeze” and “multiple trigger wheeze.” It has been suggested that the former phenotype is transient and that children with the latter phenotype will continue to wheeze and will develop asthma. However, up to now, this still needs to be confirmed. For therapeutic purposes these phenotypes may offer a practical approach in daily clinical life. Multiple trigger wheezing is far more likely to respond to treatment with an inhaled steroid than episodic viral wheeze. The ERS task force advises not to use the term “asthma” in children with preschool wheeze.

In many school-aged children, the management of asthma with occasional bronchodilator use and low- or moderate-dose inhaled corticosteroid is uncomplicated. However, problematic severe asthma is seen in a small subpopulation of asthmatic school children. This is a heterogeneous group that requires a specific and stringent work-up. The group consists of children with an incorrect diagnosis of asthma, children with asthma in addition to another disease, children with difficult asthma, and children with therapy resistant asthma.

Inhaled corticosteroid treatment is the cornerstone of preschool wheeze and asthma therapy in school children. All children should have a short-acting *β*_2_ agonist on an as needed basis, as rescue therapy. Long-acting *β*_2_ agonists are not recommended in the age group of 5 years and younger, because of absence of trials and absence of safety data. GINA guideline advocates increasing the ICS doses or adding a LABA and/or montelukast if the asthma is not-well controlled on a low-to-moderate dose of ICS. The evidence for these steps is limited. Only one study suggests starting with the addition of LABA because this appeared to be effective in most children, but many children benefit from a doubling of the ICS dosage and benefit from the addition of montelukast. This suggests that clinicians should try to individualize therapy.

A further improvement in asthma care may be achieved through improvement of adherence to guidelines by health care professionals. Therefore, implementation plans should be developed, which contain interactive and continuous education, including discussion of evidence, local consensus, feedback on performance and the making of personal and group learning plans.

## Figures and Tables

**Table 1:** Characteristics of episodic viral wheeze and of multiple trigger wheeze.

	Episodic viral wheeze	Multiple trigger wheeze
Definition	Wheezing during discrete time periods, often in association with clinical evidence of a viral cold	Wheezing that shows discrete exacerbations but also symptoms between episodes

Triggers	Viral infections	Viral infections, tobacco smoke, allergen exposure, mist exposure, crying, and exercise

Possible underlying factors	Preexistent impaired lung function, tobacco smoke exposure, prematurity, and atopy	Eosinophilic inflammation?

Continuing treatment with ICS	Little or no benefit	Significant fewer days with symptoms

Treatment with montelukast	Moderate benefit	Moderate reduction in exacerbations

Long-term outcome	Declines over time (<6 yrs) may continue into school age as episodic viral wheeze and may change into multiple trigger wheeze	May continue into adulthood as asthma

**Table 2:** Modified asthma predictive index [20].

(1) A history of ≥4 periods of wheezing episodes and at least 1 physician's diagnosis	
(2) In addition the child must meet at least 1 of the major criteria and ≥2 of the minor criteria	

Major criteria	Minor criteria

(i) Parental history of asthma	(i) Allergic sensitization to milk, egg, or peanuts
(ii) Physician diagnosed allergic dermatitis	(ii) Wheezing not related to colds
(iii) Allergic sensitization to ≥1 aeroallergen	(iii) Blood eosinophils ≥ 4%

**Table 3:** Differential diagnosis of asthma at school age.

Hyperventilation and vocal cord dysfunction	
Malformations of the airway anatomy	
(Undiagnosed) cardiac anomalies	
Cystic fibrosis	
Primary cilliairy dyskinesia	
Foreign body in the airway	
Immune deficiencies	

**Table 4:** Assessment of control for children from 6 years of age, according to the GINA guidelines [1].

Assessment of current clinical control			
Characteristics	Controlled (all of the following)	Partly controlled (any measure presented)	Uncontrolled

Daytime symptoms	None (twice or less/week)	More than twice/week	Three or more features of partly controlled asthma
Limitation of activities	None	Any	
Nocturnal symptoms/awakenings	None	Any	
Need for reliever/rescue inhaler	None (twice or less/week)	More than twice/week	
Lung function (PEFR or FEV_1_)	None	<80% predicted or personal best (if known)	

**Table 5:** GINA guidelines for children of 5 years and younger.

GINA asthma management approach based on control for children 5 years and younger		
Asthma education, environmental control, as needed *β*_2_ agonists		

Controlled on as needed rapid-acting *β*_2_ agonists	Partly controlled on as needed rapid-acting *β*_2_ agonists	Uncontrolled or only partly controlled on as needed rapid-acting *β*_2_ agonists.

Controller options		

Continue as needed rapid-acting *β*_2_ agonists	Low-dose inhaled corticosteroid	Double low-dose inhaled corticosteroid.
	Leukotriene modifier	Low-dose inhaled corticosteroid plus Leukotriene modifier.

Leukotriene modifier: leukotriene receptor agonist.

**Table 6:** Asthma treatment in children older than 6 years according to the GINA guideline [1].

Step 1	Step 2	Step 3	Step 4	Step 5
Asthma education. Environmental control				
(If step-up treatment is being considered for poor symptom, first check inhaler technique, check adherence, and confirm that symptoms are due to asthma.)				

As needed rapid-acting *β*_2_ agonist				

	**Select** **one**	**Select** **one**	**To** **step** **3** **treatment** **Select** **one** **or** **more**	**To** **step** **4** **treatment** **add** **either**
Controller options	*Low dose ICS *	*Low dose ICS+ long-acting* *β*_2_ *agonist *	*Medium- or high-dose ICS+ long-acting β*_2_ *agonist *	Oral glucocorticosteroid (lowest dose)
		Medium- or high-dose ICS Low-dose ICS + leukotriene modifier	leukotriene modifier sustained release theophylline	Anti-IgE treatment
		Low-dose ICS + sustained release theophylline		

ICS: inhaled corticosteroids. Italic and Bold words refer to the recommended treatment. Alternative reliever treatments include inhaled anticholinergics, short-acting oral *β*_2_ agonist, some long-acting *β*_2_ agonist, and short-acting theophiline. Regular using of short- and long-acting *β*_2_ agonist is not advised unless accompanied by ICS.
